# *Sargassum filipendula*, a Source of Bioactive Compounds with Antioxidant and Matrix Metalloproteinases Inhibition Activities In Vitro with Potential Dermocosmetic Application

**DOI:** 10.3390/antiox12040876

**Published:** 2023-04-04

**Authors:** Yonadys Luna-Pérez, Lady Giselle Ríos-López, Elver Luis Otero-Tejada, Juan Camilo Mejía-Giraldo, Miguel Ángel Puertas-Mejía

**Affiliations:** 1 Grupo de Investigación en Compuestos Funcionales, Facultad de Ciencias Exactas y Naturales, Universidad de Antioquia, UdeA, Calle 70 No. 52-21, Medellín 050010, Colombia; 2 Grupo de Estabilidad de Medicamentos, Cosméticos y Alimentos, Facultad de Ciencias Farmacéuticas y Alimentarias, Universidad de Antioquia UdeA, Calle 70 No. 52-21, Medellín 050010, Colombia

**Keywords:** *S. filipendula*, phlorotannins, fucoidans, matrix metalloproteinases, antioxidant

## Abstract

The antioxidant and the potential inhibitory capacity of matrix metalloproteinases of the phlorotannin-type polyphenolic and fucoidan-type polysaccharides extracts obtained from the macroalga *S. filipendula* were evaluated. Through chromatographic and spectroscopic techniques, the corresponding chemical structure of compounds present in the extracts was determined. Antioxidant capacity was evaluated using the methyl linoleate model for the inhibition of lipid peroxidation, and the free radical scavenging capacity was assessed using DPPH, ABTS, ^•^OH, O_2_^•−^ methods. The matrix metalloproteinase inhibition potential was measured by collagenase and elastase inhibition tests, using epigallocatechin gallate as a positive control. The extracts exhibited a high scavenging capacity of radical species evaluated and inhibition of diene conjugate formation and thiobarbituric acid reactive substances. The results showed that the crude extracts presented dose-dependent collagenase and elastase inhibition, with IC_50_ values between 0.04 and 1.61 mg/mL. The structure of the residues of the polysaccharide was identified mainly as (1→3)-sulfated (1→3) α-l-fucopyranose at carbon 4 and residues of β-d-glucopyranose, α-d-Mannopyranose, and β-d-Galactopyranose, while in the polyphenol extract the presence of phloroglucinol was identified and the presence of eckol, bifuhalol, and trifuhalol was suggested. Our results allow us to infer that *S. filipendula* is a potential source of bioactive ingredients with antioxidant and anti-aging activity.

## 1. Introduction

*Sargassum* sp. is a macroalgae that detaches from the seabed and is dragged by the natural movement of the sea towards the beaches, forming agglomerations that are important for biodiversity and productivity, in addition to attracting numerous species of fish, birds, and turtles. However, the proliferation of these algae has increased substantially in the last decade, reaching the beaches of the Atlantic Ocean and the Caribbean Sea, including those of Colombia and Mexico, which has caused significant environmental and economic damage. Its excessive growth has caused the loss of many species, blocking the passage of light to seagrasses and coral reefs that can no longer perform their usual photosynthesis. In addition, its decomposition generates gases that are harmful to human health, such as ammonia and hydrogen sulfide, the latter caused by the high sulfate content in the algae’s cell walls and perceived by the population as a nauseating odor [1].

*Sargassum* is a genus of marine macroalgae belonging to the class Phaeophyceae, also known as brown algae, due to the color conferred by the carotenoid fucoxanthin, abundant in chloroplasts, combined with the green color of chlorophyll. In the marine regions of the Colombian Caribbean, 619 species of benthic macroalgae were reported, grouped into blue-green algae (Phylum Cyanobacteria), red algae (Rhodophyta), green algae (Chlorophyta), and brown algae (Ochrophyta) [2,3,4], where the species *S. polyceratium* and *S. filipendula* have the highest number of reports [3]. These species have a valuable content of very relevant compounds such as polyphenols type phlorotannin, carotenoids, and polysaccharides, with food applications and bioactivities (e.g., anti-inflammatory activity) [5]. In addition, algae may be a promising ingredient as a functional food, due to their presence in carotenoids, polyphenols, and polysaccharides with pharmacological activities, e.g., immunomodulatory, antitumor, hypoglycemic, anticoagulant, antibacterial, antioxidant, anti-aging, among others [6]. Focusing on polysaccharides and polyphenols type phlorotannins, many authors described applications, e.g., bone growth, antiviral, anti-inflammatory, immunomodulatory, anticoagulant, anticancer and antiproliferative activities [6,7] and recent studies demonstrated their potential for the prevention of premature skin aging with antioxidant and anti-enzymatic activities [8].

Fucoidans are sulfated anionic polysaccharides isolated from the cell walls of marine algae (brown algae) and their structural difficulty diverges in the degree of branching, type of functional groups, degree of sulfation, fine structure, and kind of bonds, additionally, according to their geographical origin and species, their composition also varies. In general, they are composed of sulfate groups and fucose with minor proportions of xylose, uronic acids, mannose, arabinose, and galactose [9,10]. In contrast, polyphenols are a large group of secondary metabolites that are characterized by having in their structure one or several aromatic rings mono or polysubstituted by the hydroxyl group, considered an electron donor [11]. Phlorotannins are polyphenols whose presence is mainly associated with brown algae [12]. They are oligomers of phloroglucinol (1,3,5 trihydroxybenzene) linked linearly and/or through branches. According to the type of intermolecular bond between phloroglucinol units and the presence of the additional hydroxyl group in position C2, C4, and C6, phlorotannins are subdivided into six groups: aryl-bonded fucol, ether-bonded phloretol, ether-bonded fuhalol, and additional hydroxyls, fucophloretol with the presence of aryl and ether bonds, eckol with the appearance of a dibenziodoxin ring, and carmalol with the dibenzodioxin ring and additional OH [13,14].

Premature aging of the skin is the main effect of the decrease in the primary structural components of the extracellular matrix in the dermis (collagen, elastin, and hyaluronic acid), and preventing their degradation is of vital importance, as it allows the generation of strategies that help protect the skin barrier from the signs of aging, avoid excessive dehydration, as well as protect it from ROS and ultraviolet radiation. Premature skin aging can be delayed by using mechanisms such as cosmetological care, the use of sunscreens, different anti-aging actives, such as moisturizers, hydrators, antioxidant agents, cell regulators, and enzymatic inhibitors of collagenase, elastase and/or hyaluronidase. In addition to a convenient correction of lifestyle and habits to avoid exogenous factors of aging [15].

Consequently, the aim of the research was to evaluate the potential antioxidant capacity and matrix metalloproteinase inhibition activity in vitro of phlorotannins and fucoidan extracts from the algae *Sargassum filipendula.*

## 2. Materials and Methods

### 2.1. Collection of Biological Material and Extraction Process

The algae were collected from the beaches of Taganga, Santa Marta (Magdalena, Colombia), (RGE0156-5, Otrosí # 5, Contract for access to genetic resource and its derived products, 126 of 2016, Ministry of Environment and Sustainable Development, Bogotá, Colombia). The material was rinsed and dehydrated at 50 °C for 6 h in an oven. The dried brown seaweed (DS) was added in a coffee grinder and roughly pulverized. From the ground material, the extraction of polyphenols and polysaccharides was carried out, as follows:

Fucoidan extraction: Was performed according to the method described by Kim et al. [10] with minimal changes. The previously washed, dried, and ground raw material was suspended for 24 h in an HCl solution (0.1 M) at room temperature. Subsequently, the filtrate obtained was neutralized (NaOH, 1.0 M). Then, 3 volumes of ethanol were used to precipitate the extract. The precipitate obtained was solubilized in water and the pH was corrected to 2, using HCl (1.0 M). Calcium chloride solution (4.0 M) was used to remove alginates by precipitation and, using 3 volumes of ethanol and redissolving it in water, the supernatant was precipitated. Purification of the extract enriched in fucoidan-type polysaccharides was carried out by redissolving in water and dialyzing (molecular weight cutoff: 12 to 14 kDa) at 4 °C for 72 h. Finally, the obtained enriched-crude extract was purified using an anion exchange column (DEAE cellulose, Sigma-Aldrich, Saint Louis, MO, USA) with a gradient of NaCl solution (0.5, 1.0, 1.5, 2.0, 2.5, and 3.0 M at pH 2.0); subsequently, this additional fraction, termed HiP1, was subjected to dialysis for 24 h and lyophilized. All fractions were stored at room temperature for subsequent analysis.

Phlorotannins extraction: The previously washed, dried, and ground raw material was extracted by successive extractions for 12 h in acetone with magnetic stirring at room temperature. Then, it was dried in a rotary evaporator (IKA, RV10 basic, IKA, Campinas, Brazil) at 40 °C.

### 2.2. Sugar Content Analysis of Fucoidan Extracts

#### 2.2.1. Neutral Sugars Content

The phenol-sulfuric acid method was used to quantify neutral sugars [16]. Samples and reference substances of fucose were added to 2.0 mL of H_2_SO_4_ (37% *v*/*v*) and subjected to magnetic stirring for 15 s at 4000 rpm. Then, the final solution and 400 µL of phenol (5% *v*/*v*) were mixed and heated at 90 °C for 5 min, and then, chilled in a water bath. The absorbance of fucose was read at 480 nm, respectively, using a UV-Vis spectrophotometer (Evolution 60S, Thermo Fisher Scientific, Inc., Shanghai, China). The content of neutrals sugars was determined using Equation (1)

(1) $$\%\;Neutral\;sugar\;\left(fucose\right)=\left(\frac{A_{sample}+0.0050}{0.0028}\right)\times \left(\frac{400\mu L}{V_{sample}\times C_{sample}}\right)\times 100$$

where, A_sample_ = Sample’s absorbance at 490 nm; V_sample_: Sample’s volume added in each assay, in µL; C_sample_: initial concentration of the sample in µg/mL.

#### 2.2.2. Acid Sugars Content

A total of 0.090 mL of each sample was mixed with 0.4 mL of deionized water, based on the acid method [16]. The reference substance, d-glucuronic acid, and samples were mixed with sodium tetraborate (2 mL, 0.95 g/L sulfuric acid) and heated for 12 min at 100 °C. Afterward, carbazole (0.040 mL, 0.2% *w*/*v*, in ethanol) was added and continued heating for 10 min at 100 °C. Finally, the absorbance was read at 525 nm, using a UV-Vis spectrophotometer (Evolution 60S, Thermo Fisher Scientific, Inc., Shanghai, China). The content of acid sugars was reported in terms of hexuronic acid according to Equation (2):

(2) $$\%\;Hexuronic\;acids=\left(\frac{A_{sample}+0.0144}{0.0145}\right)\times \left(\frac{400\mu L}{V_{sample}\times C_{sample}}\right)\times 100$$

#### 2.2.3. Sulfated Sugar Content

The sample (0.04 mL, at 1 mg/mL) was mixed with deionized water (0.5 mL). Then, chondroitin sulfate, samples and DMMB solution (4 mL, prepared by mixing 1 L of sodium acetate (0.05 M, pH 4.75) with 11 mg of 1,9 dimethylmethylene blue) were mixed. The mixture was subjected to magnetic stirring for 15 s at 4000 rpm and allowed to react protected from light for 30 min. The absorbance was read in a UV-Vis spectrophotometer, at 520 nm [17]. (Evolution 60S, Thermo Fisher Scientific, Inc., Shanghai, China). The percentage of sulfated sugars was obtained by Equation (3):

(3) $$\%\;Sulfated\;sugars=\left(\frac{A_{sample}+0.0058}{0.0064}\right)\times \left(\frac{500\mu L}{V_{sample}\times C_{sample}}\right)\times 100$$

### 2.3. Antioxidant Properties of Fucoidan and Phlorotannins Extracts

#### 2.3.1. Quantification of Total Phenolic Content

The phenolic content (TPC) was evaluated by the colorimetric Folin–Ciocalteu [18] method with small modifications. Succinctly, extract solution (100 µL) and deionized water (1.6 mL) were added to a reaction tube. Then, the Folin-Ciocalteu reagent (0.1 mL) was added to the solution and left to react for 9 min. Next, sodium carbonate solution (0.3 mL at 20% *w*/*v*) was added into the reaction tubes and mixed. The TPC was obtained using Equation (4) after reading the absorbance at 765 nm, in an Evolution 60S spectrophotometer (Thermo Fisher Scientific, Inc., Shanghai, China).:

(4) $$\%\;TPC\;(w/w)=\left(\frac{A_{sample}+0.0038}{0.0799}\right)\times \left(\frac{1600\mu L}{V_{sample}\times C_{sample}}\right)\times 100$$

The results are expressed as milligrams of Gallic acid equivalents per g dry extract (GAE g^−1^ DE) and percentage (milligrams of Gallic acid equivalents per 100 milligrams dry extract).

#### 2.3.2. In-Vitro ABTS Radical Scavenging Method

The ABTS (2,2′-azinobis-(3-ethylbenzothiazoline-6-sulfonic acid, ABTS) assay was performed according to a modified methodology previously described by Hamed et al. [19]. Briefly, ABTS in solution (7.0 mM) and potassium persulfate (2.45 mM) were mixed and left protected from light for 12 h, at room temperature. Then, the ABTS radical formed was mixed with distilled water until obtain an absorbance of 0.70 ± 0.02 at 734 nm. Thereafter, 0.01 mL of an appropriate dilution of the sample was added to 1.990 mL of the ABTS radical solution. After 6 min of reaction, the absorbance was read (at 734 nm), in an Evolution 60S spectrophotometer (Thermo Fisher Scientific, Inc., Shanghai, China). The results were expressed in terms of EC_50_ (the concentration (EC_50_) at which the DPPH concentration was reduced by 50%). As reference substances, t-butylhydroxytoluen (BHT) and ascorbic acid were used. The scavenging capacity of the samples was obtained by Equation (5):

(5) $$\%\;ABTS\;scavenging\;capacity=\left(\frac{A_{ABTS+\cdot }-A_{sample}}{A_{ABTS+\cdot }}\right)\times 100$$

#### 2.3.3. DDPH Scavenging Activity Assay

0.2 mL of each sample and 1.0 mL of DPPH (50 μM, 2,2-Diphenyl-2-picrylhydrazyl, (DPPH) stable radical) were mixed in methanol and subjected to mechanically stirring at room temperature for 45 min [20]. After that, the absorbance was measured at 514 nm in a UV-Vis spectrophotometer (Evolution 60S, Thermo Fisher Scientific, Inc., Shanghai, China). The concentration (EC_50_) at which the DPPH concentration was reduced by 50% was given as mg of dry material/mol of DPPH radical, based on Equation (6):

(6) $$\%\;DPPH\;scavenging\;capacity=\left(\frac{A_{ABTS+\cdot }-A_{sample}}{A_{ABTS+\cdot }}\right)\times 100$$

#### 2.3.4. Hydroxyl Radical Scavenging Activity Assay

The assay was performed according to a modified methodology previously described by Gu et al. [21] and was executed by the Fenton method. Briefly, 0.2 mL of each sample was mixed with 1 mL of FeSO_4_ (0.15 mM), 0.4 mL of salicylic acid—ethanol (2 mM), 1 mL of H_2_O_2_ (6 mM, and 0.4 mL distilled water. After that, the mixed solutions were incubated at 37 °C in a water bath for 1 h, measured at 510 nm. Distilled water was used as control. The results were expressed in terms of EC_50_ (the concentration at which the ^•^OH concentration was reduced by 50%). As reference substances, ascorbic acid was used. The scavenging capacity of the samples was obtained by Equation (7):

(7) $$\%\;Hydroxyl\;radical\;scavenging\;capacity=1-\left((A-B)/C\right)\times 100$$

where:

A: Sample with H_2_O_2_.
B: Sample without H_2_O_2_.
C: Control sample.

#### 2.3.5. Superoxide Radical Scavenging Activity Assay

The assay was performed according to the methodology previously described by Shang et al. [22] with minor modifications: 0.2 mL of each sample was mixed with 4.5 mL of Tris–HCl buffer (50 mM, pH = 8.2) and incubated at 25 °C for 20 min. Briefly, 1 mL of pyrogallol (25 mM) was reacted and mixed at the same temperature for 4 min. Then, to stop the reaction immediately was added 1 mL of 8 mM HCl was added. The absorbances were measured at 325 nm. Distilled water was used as control and as reference substances ascorbic acid was used. The superoxide radical anion (O_2_^•−^) inhibition was calculated using Equation (8) and the results were expressed in terms of EC_50_.

(8) $$\%\;Superoxide\;radical\;scavenging\;capacity=1-\left((A-B)/C\right)\times 100$$

where:

A: Sample with pyrogallol.
B: Sample without pyrogallol.
C: Control sample.

#### 2.3.6. Evaluation of the Lipid Peroxidation of Methyl Linoleate (MeLo) In Vitro

The capacity to inhibit the lipid peroxidation (thiobarbituric acid reactive substances, TBARS, and conjugated diene hydroperoxide, CDH) was measured in a methyl linoleate model according to the methodology previously described [23] with some modifications. Three different solutions, negative control (MeLo, 10 mM), positive control (0.9 mL of MeLo (10 mM), 0.1 mL of BHT (butyl hydroxytoluene, 0.2% *w*/*v*)), and extracts solutions (consisting of 0.9 mL of MeLo (10 mM) + 0.1 mL of extracts and/or standards, 0.2% *w*/*v*) were prepared. All solutions were submitted to accelerated oxidation (40 °C) for five days. After the oxidation procedure, the samples were dissolved in ethanol (1 mL). Then, the samples were mixed with ethanol (1:25 ratio, sample:ethanol), and the absorbance was read at 234 nm for conjugated diene hydroperoxide. The quantification of CDH formed during the oxidation process was calculated using a value of 29,000 M^−1^cm^−1^, as the extinction coefficient and the results were indicated in terms of mmol CDH kg^−1^ MeLo [24]. Additionally, the malondialdehyde (MDA) content was expressed in terms of TBARS. Briefly, sample (0.4 mL), BHT (0.1 mL at 0.2% *w*/*v* in ethanol), TBA (0.5 mL at 0.37% (*w*/*v*, in HCl 0.25 mM)) and ethanol (1.0 mL), were added in a reaction tube. The final solution was heated in a lab oven (Memmert lab oven, UF55) for 30 min at 90 ± 5 °C. Next, the mixture was cooled in an ice bath. After that, the flocculent precipitate was removed by centrifugation for 10 min at 140 g. For all samples, the absorbance at 535 nm was read and the absorbance read at 600 nm was subtracted to correct for nonspecific turbidity. The degree of peroxidation is given as mmol MDA kg^−1^ MeLo, using 156,000 M^−1^cm^−1^, as the molar extinction coefficient.

### 2.4. Determination of Matrix Metalloproteinases (MMPs) Inhibition Potential of Fucoidan and Phlorotannins Extracts

#### 2.4.1. Anti-Collagenase Activity

This assay was performed according to the method proposed by Widowati et al. [25] with modifications. Collagenase from *Clostridium histolyticum* type I (SIGMA-C0130, Roche Diagnostics GmbH, Mannheim Germany) at 6.25 mg/mL was prepared in Tricine buffer (50 mM, pH 7.5, and the buffer consisting of NaCl (400 mM) and CaCl_2_ (10 mM)). Then, extracts (0.150 mL), Tricine buffer (0.3 mL), and a solution containing 2 units/mL of collagenase, were mixed. Epigallocatechin-3-gallate (EGCG) was the control. To initiate the reaction, the substrate was added to the latter solution which was previously incubated at 37 °C for 20 min. After that, 0.1 mL of FALGPA, (*N*-(3-[2-Furyl]acryloyl)-Leu-Gly-Pro-Ala, 1.0 mM, in buffer) substrate, was added and the absorbance was read at 335 nm. Thereafter, the mixture is left in incubation at 25 °C for 10 min, and the absorbance (335 nm) was finally measured again. The instrumental blank was water and another blank was defined as enzyme blank containing the tricine buffer solution and the enzyme was used, in addition, the negative control consisted of the buffer solution, the substrate, and the enzyme, where total hydrolysis or higher was presented. The inhibitory potential was determined by using Equation (10) and the results were expressed in terms of IC_50_ (the concentration at which the substrate concentration was reduced by 50%).

(9) $$\%\;Inhibitory\;activity=\left(\frac{A_{NC}-A_{BE}}{A_{Sample}-A_{BE}}\right)\times 100$$

where:

A_NC_: Absorbance of the negative control after incubation.
A_BE_: Absorbance of the enzyme blank (buffer solution and enzyme).
A_Sample_: Absorbance of the sample after incubation.

#### 2.4.2. Anti-Elastase Activity

This assay was performed according to the method proposed by Shanura Fernando et al. [26] with some modifications. Elastase from porcine pancreas type I (SIGMA-E1250, Sigma-Aldrich, Saint Louis, MO, USA) at 0.43 mg/mL (4.6 U/mg) was prepared in Tris-HCl buffer (10 mM, pH 8.0). Briefly, 0.3 mL of Tris-HCl buffer was mixed with 0.075 mL of N-Succinyl-Ala-Ala-Ala-p-nitroanilide substrate (2.0 mM, in buffer). Then, 0.150 mL of each sample and 0.075 mL of elastase solution were added and mixed. The mixture is then left to incubate at 25 °C for 20 min and finally, the absorbance was measured (410 nm). The instrumental blank was water and another blank was defined as enzyme blank containing the Tris-HCl buffer solution and the enzyme was used, in addition, the negative control consisted of the buffer solution, the substrate, and the enzyme, where total hydrolysis or higher was presented. The inhibitory potential was determined by using Equation (9) and the results were expressed in terms of IC_50_ (the concentration at which the substrate concentration was reduced by 50%). 

### 2.5. Spectroscopic Characterization

Fourier Transform Infrared (FTIR-ATR), Ultraviolet-Visible (UV-Vis), and Nuclear Magnetic Resonance (NMR) spectroscopies were used to characterize the crude extracts and the purified fraction of polysaccharide extract. For FTIR experiments, a Perkin-Elmer spectrometer (Spectrum Two) equipped with an ATR accessory, with an L160000A detector (standard high-performance room temperature Lithium Tantalum with an SNR at 9300:1) and Spectrum 10 software, were used. The dry sample was pressed onto the diamond crystal, and the spectra were plotted from 4000 to 500 cm^−1^, with 28 scans at a resolution of 4 cm^−1^. The UV-Vis spectra (200 to 700 nm) were performed from the aqueous solutions of the fucoidan extract obtained and the methanolic solution of the polyphenols extract obtained. It was recorded every 1 nm with a Thermo Scientific Evolution 60S spectrophotometer, using a quartz cell with an optical length of 1 cm. NMR analysis (^1^H, ^13^C, DEPT-135, and HSQC) was recorded on an instrument Ascend 600 (Bruker) operating at 600 MHz for ^1^H and 150 MHz for ^13^C. The sample of fucoidan extract was dissolved in deuterium oxide (D_2_O) and the sample of polyphenol extract in deuterated methanol (MeOD). The ^1^H, ^13^C, DEPT-135, and HSQC spectra were taken with HOD presaturation suppression at 30 °C, using deuterated acetone as the internal standard at 2.04 ppm for ^1^H.

#### 2.5.1. Analysis of Monosaccharides by HPLC-UV

The monosaccharide composition of the CEF was analyzed by high-performance liquid chromatography (HPLC) according to the methodology proposed by Siu et al. [27] with slight modifications. An amount of 5 mg of extract was hydrolyzed with 2 mL of 2 M trifluoroacetic acid (TFA) at 110 °C for 4 h. The hydrolysate was then dried at 50 °C followed by methanol washes to remove excess TFA. The hydrolysate was redissolved in 2 mL of water. Then, a 450 µL aliquot was mixed with 450 µL of PMP (1-phenyl-3-methyl-5-pyrazolone) solution (0.5 M in methanol) and 450 µL of NaOH solution (0.3 M), and the mixture was incubated at 70 °C for 30 min. Subsequently, the reaction was neutralized with 450 µL of HCl solution (0.3 M) and partitioned three times with chloroform. The aqueous solution was collected, membrane filtered (0.45 µM), and analyzed on a 300 HPLC System liquid chromatograph equipped with a UV-VIS diode array detector (DAD). The separation was performed using a Pinnacle C18 column (5 µm 250 mm × 4.6 mm) and 17% (*v*/*v*) acetonitrile as mobile phase and the detection wavelength was 250 nm. Glucuronic acid, arabinose, galactose, glucose, fucose, and mannose were used as reference standards (Sigma, St. Louis, MO, USA).

#### 2.5.2. Analysis of Phlorotannins by HPLC-ESI-MS

The analysis of the main components present in the crude polyphenol extract was performed on a UHPLC ultimate 3000 (ThermoScientific, Waltham, MA, USA) equipped with a diode array detector (DAD) coupled with mass spectrometry. Reverse phase separation was performed on a 4.6 mm × 75 mm Symmetry column, particle size 3.5 µm, and a flow rate of 1 mL/min with gradient elution. The gradient program was: 30% B (1.4 min), 30–90% B (18.2 min), 90–30% B (2.8 min), and 30% B (2.8 min). The mobile phase was composed of (A) 0.1% (*v*/*v*) formic acid in water and (B) 0.1% (*v*/*v*) formic acid in methanol. The diode array detector was set to an acquisition range, at wavelengths between 190 nm and 700 nm. Mass analysis was performed by ESI-MS in positive ion mode (ThermoScientific LCQ Fleet ion trap, ThermoScientific, Waltham, MA, USA). Capillary voltage was set at 35 V, spray voltage was 4.5 kV, Sheath Gas (nitrogen) flux was 45 (arbitrary units) and Aux Gas was 5 (arbitrary units). The capillary temperature was 300 °C. Data were acquired over a mass range of 100–1000 *m*/*z*.

### 2.6. Statistical Analysis

The results were expressed as the means ± standard deviation (SD). All data were analyzed by one-way analysis of variance (ANOVA) followed by Tukey tests when appropriate, using R Development Core Team (2011), R: A Language and Environment for Statistical Computing, Statgraphics Centurion XIX y Microsoft Excel (Microsoft, Redmond, WA, USA). *p* values less than 0.05 (*p* < 0.05) were considered significant.

## 3. Results and Discussion

### 3.1. Yield Extraction and Proximate Composition of Fucoidan and Phlorotannin Extract

The yield for polyphenols type-phlorotannin polyphenols crude extract (CEP) and fucoidan crude extract (CEF) was 0.70 ± 0.03 and 1.85 ± 0.03% (*m*/*m*) respectively. These results were higher than those obtained by Arunkumar et al. [28] for the species *S. tenerimum* and *S. vulgare*. The CEF presented higher content of neutral and sulfated sugars than CF (*Fucus vesiculosus* extract), although, the HiP1 fraction exhibited the highest content of acid sugars (Table 1). Considering that fucoidans are polysaccharides based mainly on sulfated L-fucose, a directly proportional relationship between sulfated and neutral sugars was observed.

### 3.2. Spectroscopic Characterization of Fucoidan and Phlorotannin Extracts

#### 3.2.1. FTIR-ATR Characterization

**Figure 1:**
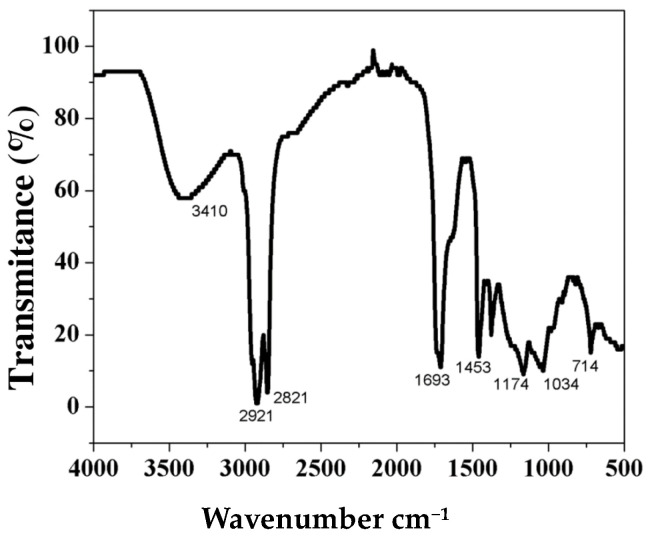
FTIR-ATR spectra of the type-phlorotannin polyphenol crude extract from *S. filipendula*.

The FTIR-ATR spectra of CEP are shown in Figure 1 with characteristic signals of the aromatic compounds. A broad band at 3410 cm^−1^ was attributed to the stretching vibration of the O-H group, and the bands at 2921 and 2821 cm^−1^ were due to C-H stretching. In addition, signals specific to aromatic rings were observed, such as the band at 1693 cm^−1^ corresponding to C=C stretching, the signal at 1453 cm^−1^ is due to C-C stretching and, finally, the signal at 1034 cm^−1^ is attributed to C-O stretching. All these bands show the presence of polyphenols in the extract obtained [29,30,31].

**Figure 2:**
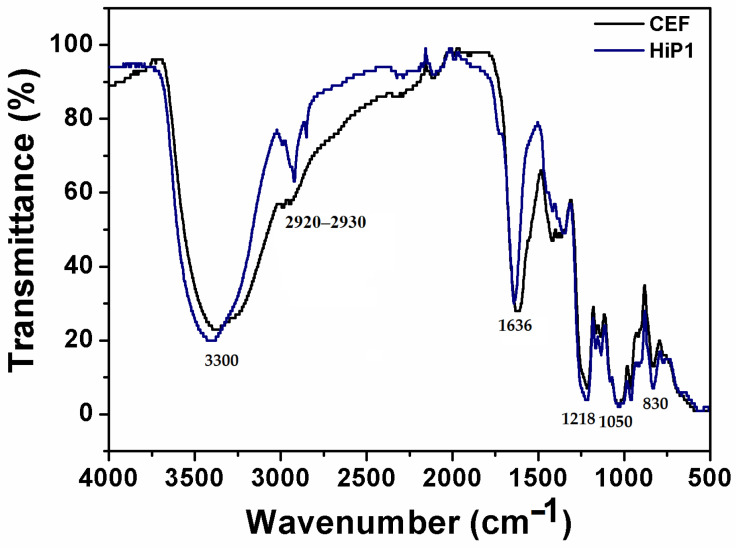
FTIR-ATR spectra of the type-fucoidan polysaccharide crude (CEF) and purified (HiP1) extracts from *S. filipendula*.

In contrast, FTIR-ATR spectra of the CEF and HiP1 are shown in Figure 2. All the spectroscopic patterns were similar, with characteristic signals of the sulfated polysaccharides. A broad band corresponding to the O-H extension vibration between 3600 and 3000 cm^−1^ characteristic of the hydroxyl group appears. Symmetric and asymmetric extension of the sp3 hybridized carbon–hydrogen (C-H) bonds is observed with a weak band between 2920 and 2930 cm^−1^ [32,33,34]. A strong vibrational band was observed between 1100 and 1000 cm^−1^, corresponding to the C-OH bond, as well as the characteristic vibrational bands of the C-O-C glycosidic bonds. In addition, the absorption bands at 1218 cm^−1^, (S=O group) and at 830 cm^−1^ (C-O-S bond) allowed us to determine the presence of the sulfate group. Finally, the presence of the carboxyl groups in the acid sugars was identified by the absorption band at 1636 cm^−1^ [35,36,37].

#### 3.2.2. NMR Characterization

The low resolution of the ^1^H-NMR spectra of the CEF and HiP1 (Figures S1 and S2) was associated with the complexity and heterogeneous characteristics of the polysaccharides, evidenced by signal overlap. However, chemical shifts characteristic of fucoidan compounds were identified (Table 2). The α-L-fucopyranose residues were identified by the presence of signals between 1.0 and 1.5 ppm, corresponding to the H6 of the methyl group. The chemical shifts of the H2, H3, H4, and H5 protons of the heterogeneous sugar residues were assigned to signals between 3.0 and 4.3 ppm. The anomeric protons (H1) were assigned to the signals at 5.0 and 5.5 ppm. Additionally, the ^13^C NMR spectra showed typical characteristic signals of α-L-fucopyranosides for the anomeric carbon (C1) between 97 and103 ppm, as well as for the methyl carbon (C6) between 15.5 and 17.0 ppm. Moreover, the heterogeneous sugar residues were associated with signals between 61 and 85 ppm, attributed to C2, C3, C4, and C5 carbons [38]. Based on the DEPT 135, ^13^C spectra, and HSQC spectra (Figures S3–S5), five different residues were recognized: (1→3) α-l-fucopyranose sulfated at carbon C4 (A) [39], (1→4) β-d-glucopyranose (B) [40], (1→3) α-l-fucopyranose without sulfate groups (C) [41], (1→2)-α-d-Mannopyranose [42] and (1→3)-β-d-Galactopyanose [43,44] (Table 2).

The ^1^H nuclear magnetic resonance analysis for the polyphenolic extract of *S. filipendula* is shown in Figure S6. Signals between 5.34 and 7.40 ppm correspond to aromatic protons; in addition, signals between 7.07 and 8.13 ppm are assigned to hydroxyl protons characteristic of polyphenols, phlorotannin type [31,45,46]. Signals between 2 and 3 ppm are assigned to the presence of acetyl groups in polyphenolic extracts of algae [47]. In contrast, the ^13^C spectrum (Figure S7) shows characteristic signals of phlorotannins. Signals between 85 and 165 ppm are typical of compounds with aromatic rings. Signals between 86 and 93.35 ppm were assigned to C-H methine carbons (C2, C4, C6); signals between 96.98 and 102.41 ppm were also observed, indicating the presence of C-C aryl bonds. Signals between 124.46 and 129.49 ppm are characteristic of C-O-C ether bonds [45]. Additionally, signals were observed at 149.98 and 150.85 ppm attributed to the presence of additional hydroxyl groups at the C2, C4, and C6 positions. Finally, signals between 151.58 and 158.59 ppm are characteristic of substituted oxygen carbons (C-OH) of C1, C3, and C5 [48,49]. According to Shrestha et al., 2021 the signals in the ^1^H spectrum at 5.77 and 5.80 ppm correspond to aromatic hydrogens, and the signals in the ^13^C spectrum at 86.17, 87.06, 88.27 ppm (C-2, C4, C6) and 150.1, 150.60, 150.85 ppm (C1, C3, C5) to phloroglucinol carbons, which is the base unit of the phlorotannins [45,46].

#### 3.2.3. Analysis of Monosaccharides by HPLC-UV

**Figure 3:**
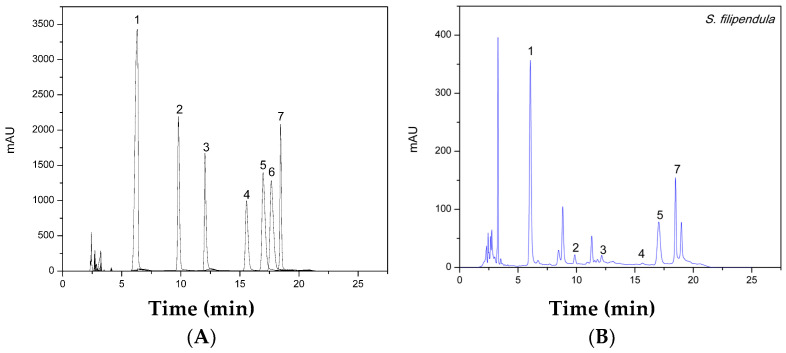
HPLC profile of monosaccharide standards (**A**) and hydrolyzed polysaccharide crude extract from *S. filipendula* (**B**). 1: PMP. 2: Mannose. 3: Glucuronic acid. 4: Glucose. 5: Galactose. 6: Arabinose. 7: Fucose.

The analysis of monosaccharide composition in the CEF was performed based on pre-column derivatization with PMP (retention time Tr 6 min). Figure 3A shows the chromatogram of the monosaccharide standards; mannose (Tr: 9.5 min), glucuronic acid (Tr: 12.3 min), glucose (Tr: 15.4 min), galactose (Tr: 17.2 min), arabinose (Tr: 17.8 min) and fucose (Tr: 18.2 min). In chromatogram B obtained for the hydrolyzed polysaccharide extract, the presence of mannose, glucuronic acid, glucose, galactose, and fucose, identified according to their Tr, together with other peaks corresponding to unidentified compounds, is observed. The results obtained in this work agree with those reported by Costa et al. [50,51] and allow us to suggest the presence of a heterofucan in *S. filipendula*.

#### 3.2.4. Analysis of Phlorotannins by HPLC-ESI-MS

The HPLC-ESI-MS analysis suggested the identification of three possible compounds in *S. filipendula* (Table 3) whose parent ion according to the literature corresponds to phlorotannins. Compound 1 (Tr 8.4 min) was identified as Eckol presenting a protonated molecular ion [M + H]^+^ at *m*/*z* 387, consistent with that reported by Yajing et al. for *S. fusiforme* [52]. Compound 2 (Tr 20.9 min) was identified as Bifuhalol with a protonated molecular ion [M + H]^+^ at *m*/*z* 267, which was reported by Zhang et al. [46] for the brown alga *Carpophyllum flexuosum*. Finally, compound 3 (Tr 26.8 min) was identified as Trifuhalol with a protonated [M + H]^+^ molecular ion at *m/z* 391, which was also reported by Phasanasophon et al., 2018 in *A. cribrosum* and Yajing et al., 2017 for *S. fusiforme* [52,53].

### 3.3. Antioxidant Activity of Fucoidan and Phlorotannins Extracts

In this work, the average total phenolic content (TPC) determined in the CEP was 32.11 ± 0.03 mg EAG/g ES, in the CEF and HiP1 were 3.21 ± 0.03 and 1.38 ± 0.12% (*w*/*w*) respectively. Regarding the screening of the antioxidant activity using the lipid model in methyl linoleate (MeLo) and radical species scavenging assays, the extracts obtained showed a significant antioxidant effect (Table 4). 

In the radical scavenging activity test, a significant difference (*p* < 0.05) was observed in all extracts with respect to the BHT standard and ascorbic acid (Table 4). The polyphenol extracts showed higher DPPH radical scavenging ability compared to fraction HiP1 and the crude extract. Boonchum et al. [54] reported an IC_50_ 45.04 mg/mL for polyphenol extract from *S. binderi* when evaluating its DPPH radical scavenging capacity, indicating a lower capacity than that obtained in this work. In contrast, Huang et al. [35] determined an IC_50_ 5.15 mg/mL for fucoidan crude extract from *S. glaucescens*, which indicates a lower antiradical activity than the one obtained in the present work, a result that is in agreement with that reported by Ye. et al. [55] in *S. pallidum*. Similarly, Dore et al. [56] reported that an acetone extract of *S. vulgare* algae, containing 2.5 mg/mL of extract presented a percentage inhibition of DPPH radicals equivalent to 22.5%. Concerning the ABTS assay, the CEF and CEP presented more potential to inhibit the ABTS radical in relation HiP1. Our results regarding the ability of the crude extract to scavenge ABTS and DPPH radicals were similar to those reported for the crude extract of the algae *S. tenerrimum* [57]. In general terms, the crude extracts showed better DPPH and ABTS radical scavenging capacity results than the purified fraction of polysaccharides. Therefore, their antiradical activity was evaluated with other radical species.

Regarding the antioxidant activity determined by the superoxide anion scavenging capacity, it was found that CEP presented an EC_50_ of 2.75 mg/mL and CEF 1.49 mg/mL with a statistically significant difference compared to ascorbic acid (*p* ˂ 0.05), with an EC_50_ of 0.47 mg/mL (Table 4). These results differ from those reported by Costa et al. [50,58] who, in a study conducted, found that *S. filipendula* collected in Brazil did not show activity against O_2_^•−^. As for the uptake capacity of the hydroxyl radical, it was determined by the Fenton reaction between hydrogen peroxide and iron, from which the ^•^OH radical was generated. The results show that the polyphenol extract of *S. filipendula* presented a lower and significantly different EC_50_ compared to the control antioxidant ascorbic acid (*p* < 0.05), it has been reported that the hydroxyl radical trapping activity of polyphenols and polysaccharides is due to their ability to chelate iron ions, resulting in metal complexes that prevent the interaction between iron and hydrogen peroxide, another mechanism is the elimination of the previously formed radical [59]. In addition, the result of antioxidant activity of the polysaccharides obtained from algae collected on the beaches of Colombia, differs, as expected from the reports of Costa et al. [50,58], since firstly, the chemical composition varies significantly from its origin and secondly, this biological material was found as marine debris, which at the time of collection was exposed to environmental conditions different from those collected directly from the seabed. Therefore, one of the objectives of this research was to verify how much biological activity could be exploited in a material naturally discarded by marine currents. 

The results obtained in this work are of great interest because superoxide anion is a precursor species of singlet oxygen, hydrogen peroxide, and the hydroxyl radical, which is why it can indirectly initiate lipid peroxidation and/or damage other macromolecules, in addition to the fact that the hydroxyl radical is considered the most reactive radical species in biological systems [51,60]. 

As for the peroxidation assay, the evaluation of accelerated oxidation of MeLo, (Table 4), demonstrated the antioxidant potential of the tested samples. Likewise, the CEP and CEF showed no significant differences (*p* > 0.05) compared to BHT and BHA in the formation of CDH and TBARS during the early and final phases of lipid peroxidation, indicating a good antioxidant capacity. Therefore, it can be inferred that the antioxidant capacity of *S. filipendula* polysaccharide and polyphenol extracts can be useful in the development of products with medical and cosmetic applications since free radicals are an important element of damage in biological systems. In general terms, *S. filipendula* could be a potentially rich source of naturally occurring antioxidant substances with promising applications in the cosmetic, food, and medicinal industries, among others.

### 3.4. Matrix Metalloproteinases (MMPs) Inhibition Potential

The results related to the matrix metalloproteinases activity showed that the polysaccharide and polyphenol extract of *S. filipendula* algae presented collagenase and elastase inhibition potential (*p* < 0.05) compared to EGCG used as a control (Table 5) and similar to that reported in the *Fucus spiralis* brown seaweed [61]. The enzyme activity for extracts and the positive control was dose-dependent. The appearance of smooth skin is mainly due to collagen and elastin fibers, two of the main components of fibrous connective tissues. However, the appearance of wrinkles in the skin is one of the most representative signs of skin aging, in response to damage to both the epidermal cells and extracellular matrix, composed mainly of elastin, collagen, and hyaluronic acid [26]. The breakdown of elastin and collagen is mainly regulated by cellular matrix metalloproteinases (MMPs), specifically, the collagenase and elastase subclasses and other types of MMPs that are involved in skin breakdown [62]. Therefore, fucoidan-type polysaccharides and phlorotannin-type polyphenols isolated from *S. filipendula* have demonstrated favorable inhibitory potential against the tested enzymes and this result allows suggesting *S. filipendula* as a primary anti-wrinkle candidate and possible source of antiaging agents.

Finally, the objective of our work which was to take advantage of the biological material of the *S. filipendula* seaweed that reaches the beaches as waste and generates environmental and economic problems, due to the affectation of tourist activities, was achieved. In this sense, although studies related to their antioxidant capacity have already been carried out, the objective of this opportunity was to give it an added value and take advantage of this material in a more complete way. Then, in addition to extracting polysaccharides, it was complemented by obtaining polyphenols and also evaluating their antioxidant capacity and their potential to inhibit metalloproteinase enzymes (collagenase and elastase). Therefore, this work becomes a chance to apply two types of extracts from the same seaweed in dermo-cosmetics, as promising ingredients with demonstrated antioxidant, anti-collagenase and anti-elastase capacity in vitro. This becomes a holistic approach to reduce the effects of age, mainly generated by oxidative and enzymatic damage to the extracellular matrix.

## 4. Conclusions

Effective separation of polysaccharides was achieved in different molecular mass ranges where the fractions obtained showed significant differences in their biological activities. Structural analysis evidenced the presence of α-l-fucopyranose type residues, with some sulfate groups and glycosidic links at C1- and C3-, as well as β-d-glucopyranose residues with glycosidic links at C1 and C4. In addition, this study demonstrated the presence mainly of polysaccharides with a molecular mass greater than 100 kDa. Furthermore, the extract has a higher content of sulfated sugars and total polyphenols compared to the commercial fucoidan of *F. vesiculosus.* The polyphenol extract showed the presence of phlorotannins identified as eckol, bifuhalol, and trifuhalol. In this sense, the antioxidant activity and matrix metalloproteinases inhibition potential of the extracts were determined in vitro and we anticipate that our approach could be useful to propose *S. filipendula* as a natural source of polysaccharides type-fucoidans and phlorotannin-type polyphenols with biological properties to develop or improve formulations with anti-aging cosmetic applications. 

## Figures and Tables

**Table 1:** Yield extraction for extracts and sugar content contents for extracts of *S. filipendula*.

Sample ^1^	Yield (%)	Fucose (% *w*/*w*)	Uronic Acids (% *w*/*w*)	Sulfated Sugars (% *w*/*w*)
CEF	1.8 ± 0.1	63.0 ± 1.1 ^a^	8.60 ± 1.5 ^a^	43.6 ± 1.8 ^a^
HiP1	17.1 ± 0.1 *	66.3 ± 1.9 ^a^	16.2 ± 0.21 ^b^	43.5 ± 1.6 ^a^
CF	-	46.9 ± 0.6 ^b^	0.24 ± 0.058 ^c^	38.2 ± 2.0 ^b^
CEP	0.7 ± 0.1	-	-	-

Results are expressed as the standard deviation of the mean value (*n* = 3). Different letters in each column indicate statistically significant differences at the 95.0% confidence level. ^1^ CEF: Type-fucoidan polysaccharide crude extract. HiP1: Purified type-fucoidan polysaccharide crude extract. * Yield extraction based on CEF. CF: Commercial fucoidan of *F. vesiculosus.* CEP: Type-phlorotannin polyphenol crude extract.

**Table 2:** Proposal structure of the residues for polysaccharides in *S. filipendula*.

Residue	Atom	Chemical Shift (ppm)					
		1	2	3	4	5	6
A: (1→3) α-l-Fuc*p*(OSO^3−^) 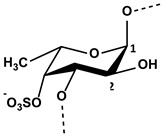	C	99.21	68.53	73.34	80.62	66.72	16.48
	H	5.26	3.96	4.22	4.63	4.38	1.17
B: (1→4) β-d-Glc*p* 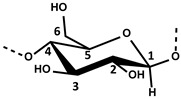	C	103.41	72.68	ND	84.52	ND	61.25
	H	4.42	3.63	ND	3.72	ND	3.74
C: (1→3) α-l-Fuc*p* 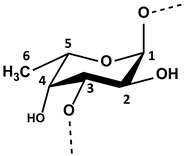	C	99.49	68.50	73.32	ND	67.42	16.49
	H	5.17	3.77	3.92	ND	4.27	1.09

**Table 3:** Phlorotannins in polyphenol crude extract from *S. filipendula*.

Compound	Suggested Florotannin	Retention Time (min)	Molecular Formula	Molecular Mass (Daltons)	Protonated Parent Ion [M + H]^+^
1	Eckol	8.4	C_18_H_10_O_10_	386.2	387.1489
2	Bifuhalol	20.9	C_12_H_10_O_7_	266.2	267.2174
3	Trifuhalol	26.8	C_18_H_14_O_10_	390.3	391.2858

**Table 4:** In vitro antioxidant activity of extracts from *S. filipendula*.

Sample ^1^	TPC ^2^ (% *w*/*w*)	mmol CDH ^3^/kg MeLo	mmol MDA ^4^/kg MeLo	DPPH Radical Scavenging Capacity, Expressed as EC_50_^5^	ABTS Radical Scavenging Capacity, Expressed as EC_50_
HiP1	1.38 ± 0.12 ^a^	ND ^6^	ND	8.19 ± 0.033 ^a^	10.1 ± 0.26 ^a^
CEF	3.21 ± 0.03 ^b^	19.97 ± 2.97 ^a^	0.28 ± 0.01 ^a^	2.90 ± 0.01 ^b^	1.86 ± 0.026 ^b^
CEP	32.11 ± 0.03 ^c^	57.46 ± 2.36 ^a^	0.50 ± 0.16 ^a^	2.83 ± 0.01 ^b^	2.90 ± 0.02 ^c^
BHT	-	31.4 ± 0.74 ^c^	0.0769 ± 0.0064 ^b^	0.571 ± 0.050 ^c^	0.377 ± 0.010 ^d^
AA	-	-	-	0.212 ± 0.0019 ^d^	0.134 ± 0.057 ^e^
MeLo	-	155.3 ± 4.5 ^d^	9.85 ± 0.28 ^c^	-	-

Results are expressed as the standard deviation of the mean value (*n* = 3). Different letters in each column indicate statistically significant differences at the 95.0% confidence level. ^1^ HiP1: Purified type-fucoidan polysaccharide crude extract, CEF: Type-fucoidan polysaccharide crude extract, CEP: Type-phlorotannin polyphenol crude extract, BHT: butylated hydroxytoluene, AA: ascorbic acid, MeLo: methyl linoleate. ^2^ TPC: total phenolic content, percentage of mg gallic equivalents per g dry extract. ^3^ CDH: conjugated diene hydroperoxide. ^4^ MDA: malondialdehyde. ^5^ EC_50_: efficient concentration at 50% (mg/mL). ^6^ ND: No activity was detected.

**Table 5:** Anti-collagenase and anti-elastase potential of *S. filipendula* extracts.

Sample ^1^	Anti-Collagenase Capacity Expressed as IC_50_ (mg/mL) ^2^	Anti-Elastase Capacity Expressed as IC_50_ (mg/mL)
HiP1	9.97 ± 0.16 ^a^	ND ^3^
CEF	1.61 ± 0.00 ^b^	0.04 ± 0.02 ^a^
CEP	0.36 ± 0.01 ^c^	0.04 ± 0.01 ^a^
EGCG	0.17 ± 0.00 ^d^	0.04 ± 0.01 ^a^

Results are expressed as the standard deviation of the mean value (*n* = 3). Different letters in each column indicate statistically significant differences at the 95.0% confidence level. ^1^ HiP1: Purified type-fucoidan polysaccharide crude extract, CEF: Type-fucoidan polysaccharide crude extract, CEP: Type-phlorotannin polyphenol crude extract, EGCG: epigallocatechin-3-gallate. ^2^ IC50: inhibition concentration at 50% (mg/mL). ^3^ ND: No activity was detected.

## Data Availability

All data generated or analyzed during this study are included in this published article (and its Supplementary Information Files).

## Supplementary Materials

The following supporting information can be downloaded at: https://www.mdpi.com/article/10.3390/antiox12040876/s1, Figure S1: NMR 600 MHz 1H spectra of the type-fucoidan polysaccharide crude extract from *S. filipendula*; Figure S2: NMR 600 MHz 1H spectra of the purified type-fucoidan polysaccharide crude extract from *S. filipendula*; Figure S3: NMR 600 MHz 13DEPT-135 spectra of type-fucoidan polysaccharide extract from *S. filipendula*; Figure S4: NMR 600 MHz 13C spectra of type-fucoidan polysaccharide extract from *S. filipendula*; Figure S5: NMR 600 MHz HSQC spectra of type-fucoidan polysaccharide extract from *S. filipendula*; Figure S6: NMR 600 MHz 1H spectra of the type-phlorotannin polyphenol crude extract from *S. filipendula*; Figure S7: NMR 600 MHz 13C spectra of the type-phlorotannin polyphenol crude extract from *S. filipendula*.
