# Supplementary material for: Sargassum filipendula, a Source of Bioactive Compounds with Antioxidant and Matrix Metalloproteinases Inhibition Activities In Vitro with Potential Dermocosmetic Application
Source: Antioxidants (Basel). 2023 Apr 4;12(4):876. doi: 10.3390/antiox12040876 (PMC10135785; doi:10.3390/antiox12040876)
Supplement: Supplementary file 1 [file antioxidants-12-00876-s001.zip › antioxidants-2265330-supplementary.pdf]

***Sargassum filipendula*, a source of bioactive compounds with antioxidant and matrix metalloproteinases inhibition activities *in vitro* with dermocosmetic application**

Yonadys Luna-Pérez[1,2], Lady Giselle Ríos-López[1], Elver Luis Otero Tejada[1], Juan Camilo Mejía-Giraldo [1,2], Miguel Ángel Puertas-Mejía[1*]

[1]*Grupo de Investigación en Compuestos Funcionales, Facultad de Ciencias Exactas y Naturales, Universidad de Antioquia, UdeA, Calle 70 No. 52-21, Medellín 050010, Antioquia, Colombia*

[2]*Grupo de estabilidad de medicamentos, cosméticos y alimentos, Facultad de Ciencias Farmacéuticas y Alimentarias, Universidad de Antioquia UdeA, Calle 70 No. 52-21, Medellín 050010, Antioquia, Colombia*

* Corresponding author: Miguel A. Puertas-Mejía. Email: miguel.puertas@udea.edu.co

ORCID of the author

Miguel A. Puertas-Mejía: 0000-0002-9962-669X

Juan C. Mejía-Giraldo: 0000-0002-2722-988X

Yonadys Luna-Pérez: 0000-0002-2133-7468

ELECTRONIC SUPPLEMENTARY MATERIAL

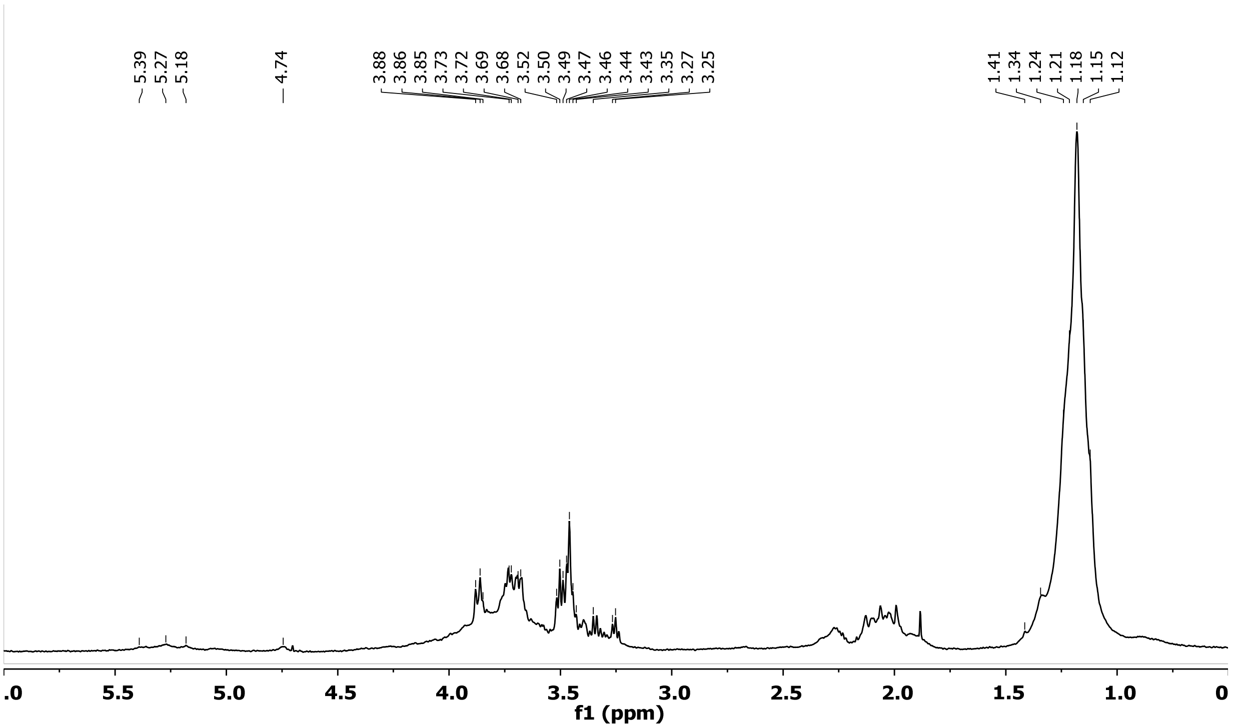

Figure S1. NMR 600 MHz ^{1}H spectra of the type-fucoidan polysaccharide crude extract from *S. filipendula*

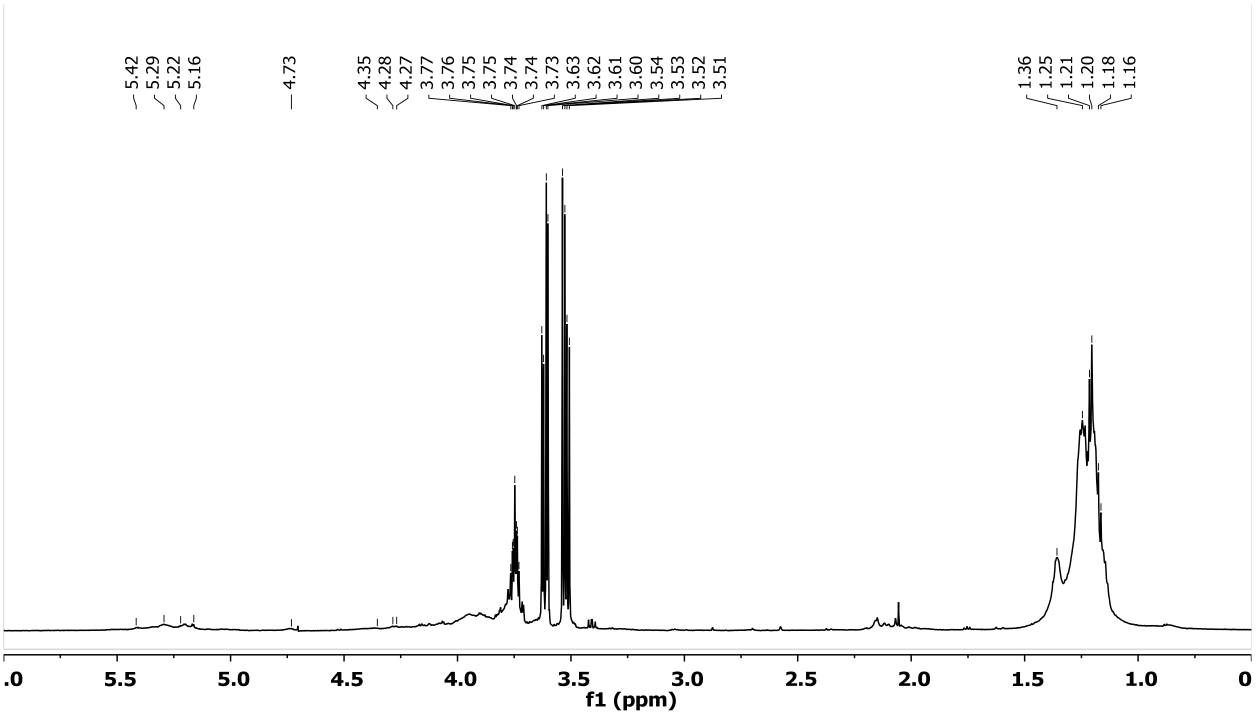

Figure S2. NMR 600 MHz ^{1}H spectra of the purified type-fucoidan polysaccharide crude extract from *S. filipendula*

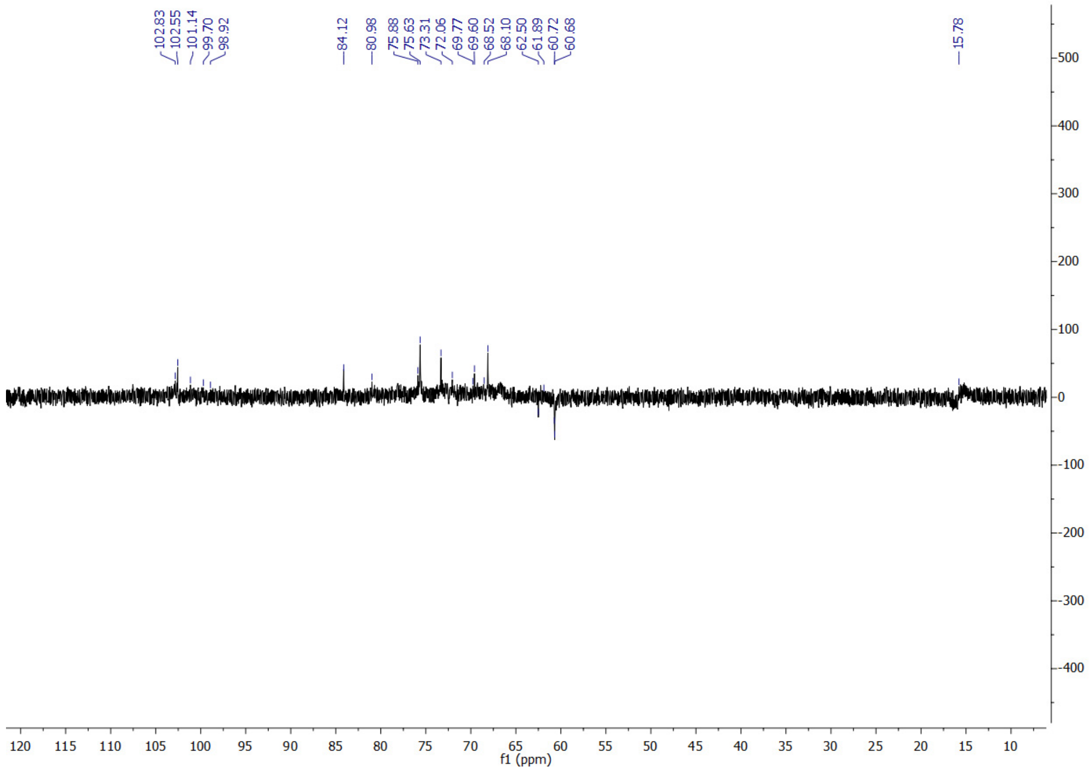

Figure S3. NMR 600 MHz ^{13}DEPT-135 spectra of type-fucoidan polysaccharide extract from *S. filipendula*

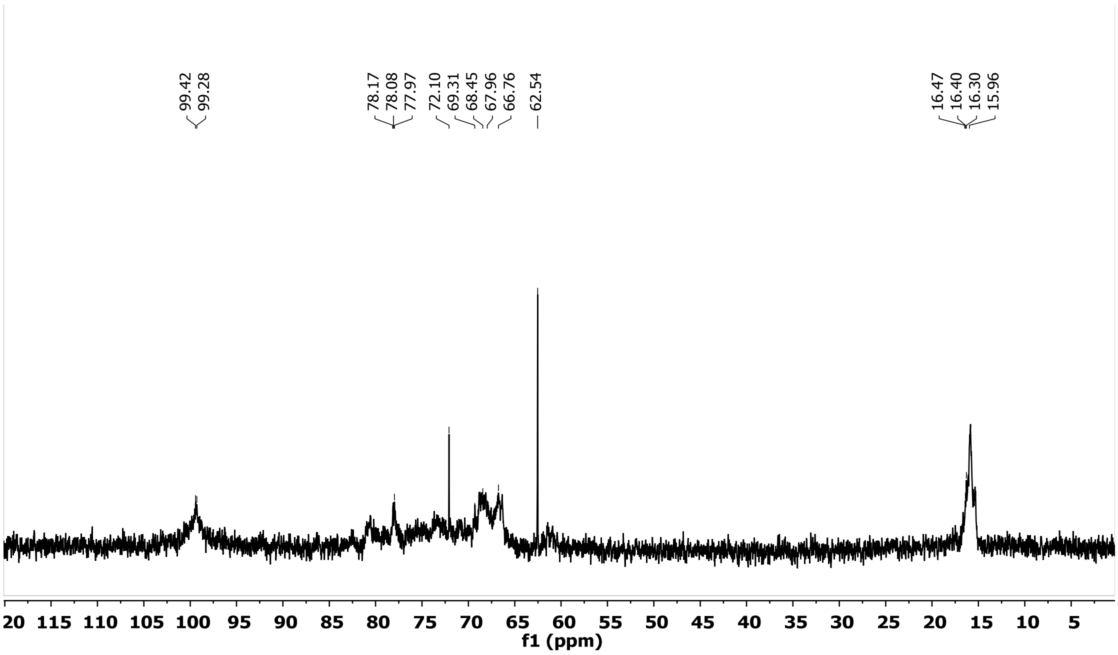

Figure S4. NMR 600 MHz ^{13}C spectra of type-fucoidan polysaccharide extract from *S. filipendula*

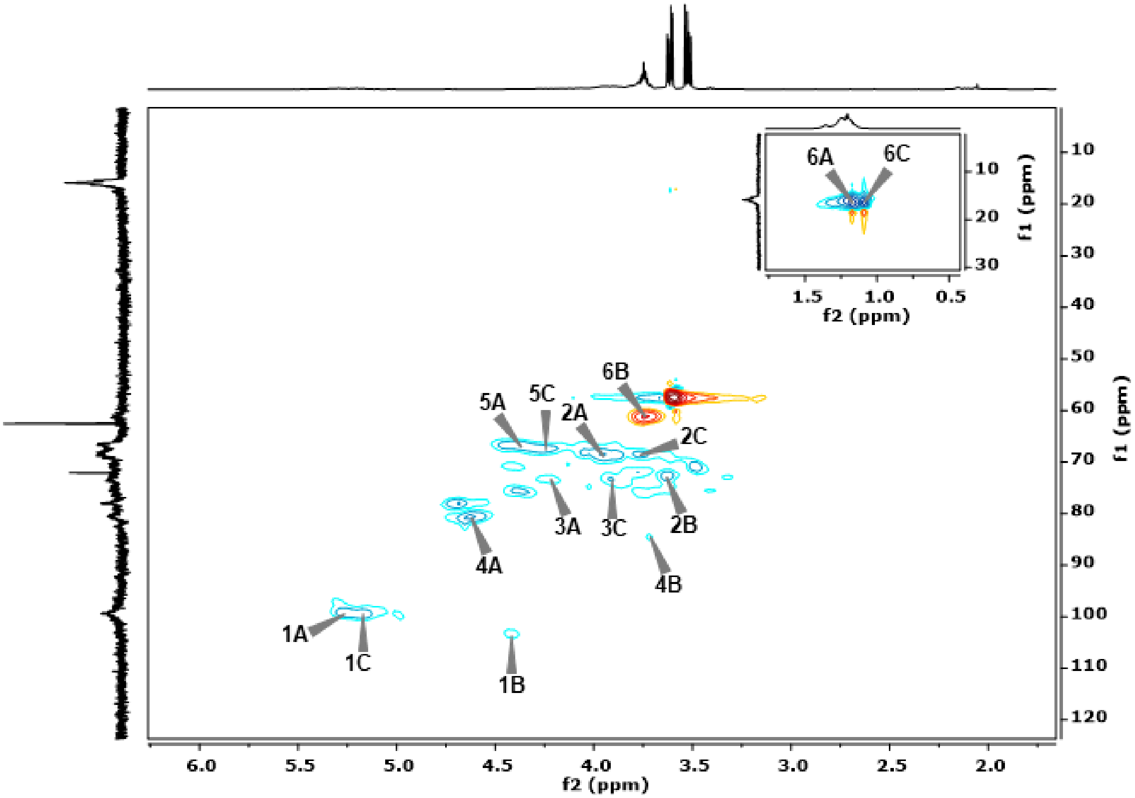

Figure S5. NMR 600 MHz HSQC spectra of type-fucoidan polysaccharide extract from *S. filipendula*

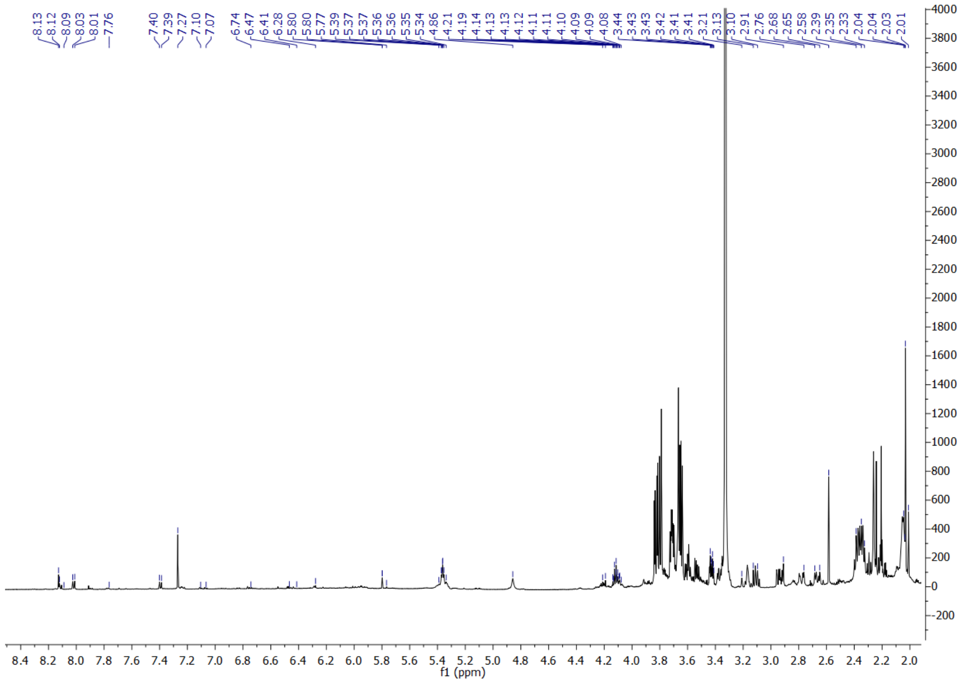

Figure S6. NMR 600 MHz ^{1}H spectra of the type-phlorotannin polyphenol crude extract from *S. filipendula*

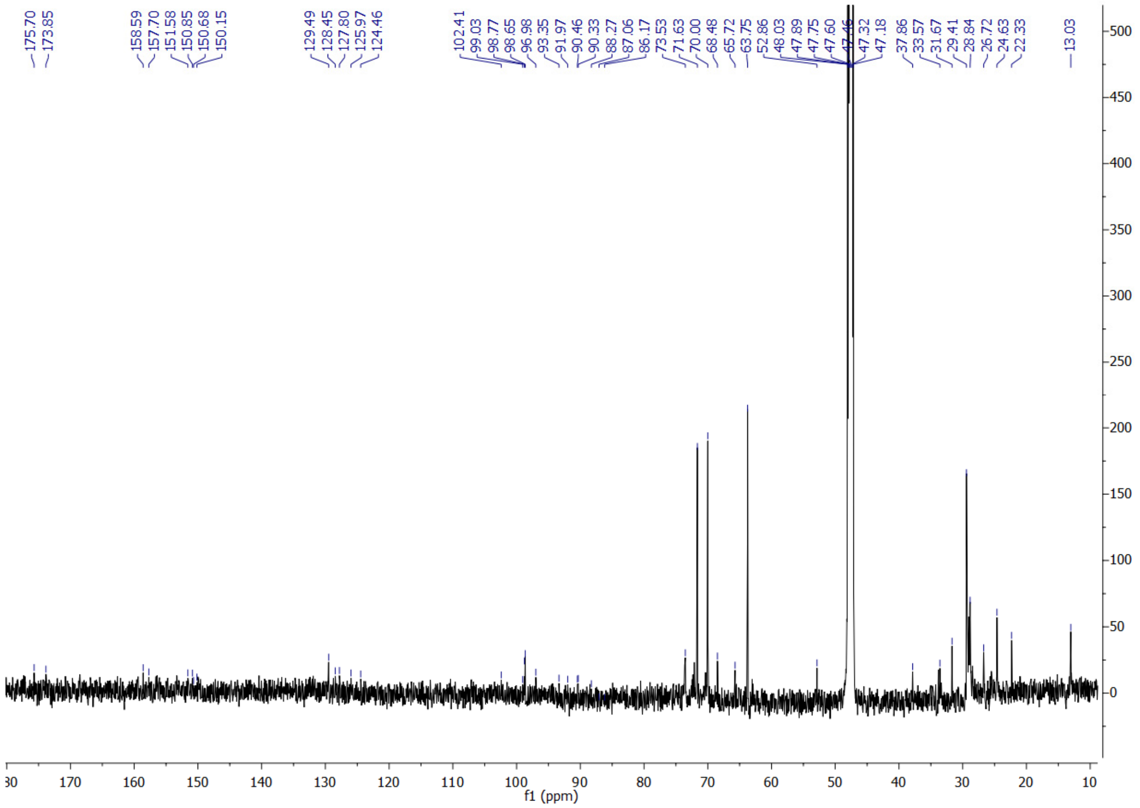

Figure S7. NMR 600 MHz ^{13}C spectra of the type-phlorotannin polyphenol crude extract from *S. filipendula*
